# 406. Incidence of Community and Hospital Acquired Infections in Critically Ill COVID-19 Patients in the Dominican Republic

**DOI:** 10.1093/ofid/ofab466.607

**Published:** 2021-12-04

**Authors:** Rita Alexandra Rojas-Fermin, Anel E Guzman, Ann Sanchez, Edwin Germosen, Cesar Matos, Alfredo J Mena Lora

**Affiliations:** 1 Hospital General Plaza de la Salud, Santo Domingo, Dominican Republic; 2 Universidad Tecnologica de Santiago, Santo Domingo, Distrito Nacional, Dominican Republic; 3 University of Illinois at Chicago, Chicago, Illinois

## Abstract

**Background:**

The disease caused by SARS-CoV-2, COVID-19, has caused a global public health crisis. COVID-19 causes lower respiratory tract infection (LRTI) and hypoxia. There is a paucity of data on bacterial and fungal coinfection rates in patients with COVID-19 at low and middle income countries (LMICs). Our objective is to describe the clinical characteristics of critically ill patients with COVID-19 in the Dominican Republic (DR)

**Methods:**

We performed a retrospective review of patients admitted to the ICU with COVID-19 from March 14th to December 31st 2020, at a 296-bed tertiary care level and teaching Hospital in the Dominican Republic. Demographic and clinical information was collected and tabulated. Laboratory confirmed bacterial and fungal infections were defined as community acquired infections (CAI) if diagnosed within 48 hours of admission and hospital acquired infections (HAI) when beyond 48 hours. Microbiologic data was tabulated by source and attribution.

**Results:**

Our cohort had 382 COVID-19 patients. Median age was 64 and most were male (64.3%) and 119 (31.1%) were mechanically ventilated and 200 (52%) had central venous catheters. A total of 28 (7%) laboratory confirmed community acquired infections and 55 (14%) HAIs occurred. Community acquired infections included 13 (46%) bloodstream infections (BSIs), 11 (39%) urinary tract infections (UTI) and 6 (21%) LRTIs. HAIs included 39 (70%) BSIs, 11 (20%) UTIs and 6 (11%) ventilator associated pneumonias (VAP). Causal organisms of community and hospital acquired BSI and UTI are in Figure 1 and Figure 2 respecively. All-cause mortality was 35.3% (135/382) in our cohort, and 100% mortality (76) in those with coinfections.

Figure 1. Community acquired and hospital acquired bloodstream infections in COVID-19 patients admitted to the ICU

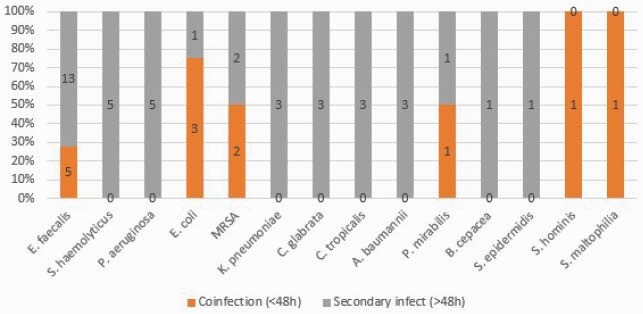

Figure 2. Community acquired and hospital acquired urinary tract infections in COVID-19 patients admitted to the ICU

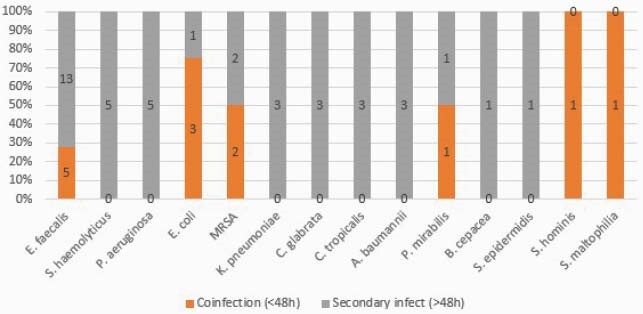

**Conclusion:**

Community and hospital acquired infections were common and in the ICU and likely contributed to patient outcomes. More than two thirds of HAIs in the ICU were BSIs. Central venous catheter device utlization and maintenance may play a role in BSIs, along with immunosuppression from COVID-19 therapeutics and translocation from mucosal barrier injury. Mortality in patients with coinfections was higher than those without. Infection prevention strategies to reduce device utilization during COIVD-19 in LMICs may have an impact on HAIs.

**Disclosures:**

**All Authors**: No reported disclosures

